# Can the Acid-formation Potential of Saliva Detect a Caries-related Shift in the Oral Microbiome?

**DOI:** 10.3290/j.ohpd.b2573053

**Published:** 2022-01-20

**Authors:** Cornelia Frese, Lisa-Sophie Reissfelder, Samuel Kilian, Anna Felten, Lutz Laurisch, Kyrill Schoilew, Sebastien Boutin

**Affiliations:** a Associate Professor, Department of Conservative Dentistry, Dental School, University Hospital Heidelberg, Heidelberg, Germany. Design, ethical approval, data analysis, drafted the manuscript.; b Dentist, Department of Conservative Dentistry, Dental School, University Hospital Heidelberg, Heidelberg, Germany. Data acquisition and analysis, critically revised the manuscript.; c Biometrician, Institute of Medical Biometry and Informatics, Heidelberg University, Heidelberg, Germany. Data analysis, critically revised the manuscript.; d Dentist, Department of Conservative Dentistry, Dental School, University Hospital Heidelberg, Heidelberg, Germany. Data acquisition and analysis.; e Dentist, Private Practice for Dentistry and Individual Prophylaxis, Korschenbroich, Germany. Study concept, critically revised the manuscript.; f Dentist, Department of Conservative Dentistry, Dental School, University Hospital Heidelberg, Heidelberg, Germany. contributed to the conception, design, ethical approval, critically revised the manuscript.; g Junior Group Leader ‘Microbiome’, Department of Infectious Diseases, Medical Microbiology and Hygiene, University Hospital Heidelberg, and Translational Lung Research Center Heidelberg (TLRC), German Center for Lung Research (DZL), Heidelberg, Germany. Microbiome and data analysis, critically revised the manuscript.

**Keywords:** acid formation, bacterial tests, caries, oral microbiome, saliva

## Abstract

**Purpose:**

To determine acid-formation potential of saliva and evaluate whether this method corresponds with microbiome composition of individuals with and without caries.

**Materials and Methods: A clinical, controlled pilot study was performed with two groups:**

individuals without caries (n = 25; DMFT = 0) and individuals with at least one active carious lesion (n = 25; DMFT>0). A detailed intraoral examination was performed, and the gingival bleeding index (GBI) and plaque index (PI) were recorded. The acid-formation potential was measured (ΔpH) after 1 h. *Streptococcus mutans* (SM) and lactobacilli (LB) were also quantified. Intergroup comparisons were made using the Mann-Whitney U-test. The diagnostic value was evaluated using the receiver operating characteristics (ROC) method and area under the curve (AUC) values were calculated. The saliva microbiome was analysed by 16S rDNA next-generation sequencing.

**Results:**

A statistically significant difference was found in ΔpH, with the ‘caries’ group showing a higher mean value after 1 h (‘healthy’ = 1.1,’caries’ = 1.4; p = 0.035). The AUC values were moderate to good (ΔpH = 0.67; SM = 0.83; LB = 0.83;1 = ideal). *Streptococcus mutans* and Lactobacilli were more frequently detected in the ‘caries’ group (p < 0.001), as were statistically significantly higher GBI (p = 0.006) and PI (p = 0.001). The saliva microbiome had a higher α-diversity and greater richness in individuals with active caries. The incidence of the genera *Alloprevotella, Prevotella, Campylobacter* and *Veillonella* was statistically significantly higher in the ‘healthy’ group. The incidence of the genera *Fretibacterium, Lactobacillus,* and *Leptotrichia,* as well as the phyla Spirochaetes and Synergistetes, was statistically significantly higher in the ‘caries’ group.

**Conclusion:**

Further studies must be carried out to determine the extent to which both tests are suitable for predicting future caries development.

Determining a shift in an individual’s oral microbiome from homeostasis to dysbiosis and therefore the increased risk of caries would be indispensable for planning preventive interventions in a timely manner so that irreversible loss of hard tooth substance can be prevented. Despite efforts to intervene early in the caries process, to date, the individual’s previous caries experience remains the most precise single parameter to predict caries risk.^9,22,27,31,36,38,44^

Saliva has been used in commercially available tests and in clinical studies to determine the risk of caries.^11,33,39^ It can be extracted easily and non-invasively at low cost, and bacterial parameters can be obtained by quantifying caries-associated species of mutans streptococci and lactobacilli. Furthermore, high-quality bacterial DNA can be extracted from saliva, making it an ideal substance for diagnostics. However, non-bacterial parameters, such as salivary flow rate, buffering capacity, and pH value, are only marginally suitable for predicting disease,^3,19^ and must be combined with other parameters, such as bacterial predictors. Although some studies have reported different microbial diversity between saliva and supragingival plaque,^13,16,34^ Shi et al^37^ showed that the salivary microbiome has positive associations with the supragingival microbiome. This indicates that saliva samples may be useful for monitoring the supraginigival microbiome. Indeed, a positive correlation was found between the numbers of mutans streptococci and lactobacilli in plaque and saliva.^23^

Oral health is reflected in the balance between host and oral microorganisms. This describes the currently most widely accepted theory of the aetiology and pathogenesis of caries: the ecological plaque hypothesis.^21^ This hypothesis combines the specific and unspecific plaque hypotheses, and a shift in the described equilibrium (dysbiosis) enables the caries process. Continuous consumption of fermentable carbohydrates increases the number of acidogenic and aciduric microorganisms in saliva, which gradually displace non-pathogenic microbiota.^43^ Consequently, these acidogenic and aciduric microorganism have the potential to change the environment in the oral cavity. The acid-formation potential of the saliva, which is measured after one hour of incubation, represents a new method that might be helpful in the detection of ecological and caries-related changes within the oral cavity.

This pilot study addresses the need for suitable test procedures and additional screening tools to intervene early in the caries process. As this is the first study set-up evaluating this new salivary parameter, we tested whether the acid-formation potential of saliva is able to discriminate between a ‘caries’ and ‘healthy’ group in accordance with data obtained from conventional bacterial tests and saliva microbiome analysis.

## Materials and Methods

This was an exploratory pilot study carried out as part of a clinical controlled cross-sectional investigation and was approved by the local medical ethics committee (S-389/2017). All patients gave written informed consent in accordance with the Declaration of Helsinki. Participants were selected for inclusion in the study based on the following inclusion and exclusion criteria:

### Inclusion criteria

- Able to give consent
- Written informed consent
- Older than 18 years
- Participants with a DMFT value of 0 were allocated to the ‘healthy’ group
- Participants with a DMFT value of > 0 and at least on cavitated carious lesion were allocated to the ‘caries’ group.

### Exclusion Criteria

Hyposalivation (unstimulated saliva flow rate below 0.1 ml/min and stimulated salivary flow rates below 0.7 ml/ min) due to:

- Systemic diseases (Sjögren’s syndrome, salivary gland diseases, diabetes mellitus, neurological diseases)
- Tumors, operations, and/or radiation in the head and neck area
- Psychological disorders
- Drugs with an anti-sialagogue effect (e.g. psychotropic drugs, appetite suppressants, antihypertensive agents, antihistamines, diuretics, cytostatics)
- Reduced chewing ability
- Use of antibiotics in the past 14 days
- Use of antibacterial mouthwash in the last 12 days
- Smoking or chewing gum within 2 h before the examination
- Eating or drinking directly before the examination.

Participants were divided into two groups (‘caries’ and ‘healthy’) according to the aforementioned criteria. In the ‘healthy’ group (n = 25), participants were naturally healthy (DMFT = 0) and had no prior history of caries or invasive restorative dental treatments. Participants with at least one cavitated caries (DMFT > 0) were allocated to the ‘caries’ group. The dental examination comprised an extraoral and intraoral examination, a sensitivity and percussion test, and systematic recording of the DMFT index. In accordance with Klein et al,^17^ we determined the DMFT index based on examination of 28 teeth. Any teeth with restorations or carious lesions were scored. Two probing depths (mesio- and disto-buccal) were measured for each tooth and the bleeding-on-probing (BOP) index was recorded. The gingival bleeding index (GBI)^2^ and the plaque index or plaque control record (PCR)^26^ were also measured.

Saliva tests were performed on stimulated saliva (patients chewed paraffin). The following were measured for every participant:

1. the acid-formation potential of saliva after 1 h of incubation
2. the rate of secretion over 5 min
3. the buffering capacity
4. the number and occurrence of mutans streptococci and lactobacilli.

### Acid-formation Potential

This novel saliva test determined the acid-formation potential or pH difference (ΔpH) of the saliva after 1 h of incubation (patent pending). The pH of the saliva was measured using McloropHast pH 6.5–10.0 pH indicator sticks (Merck; Darmstadt, Germany) before the test was carried out. One milliliter of the stimulated saliva was then pipetted into the test nutrient medium and incubated at 37°C for 1 h. Alternatively, the sample can be incubated for 1 h in the patient’s pocket while professional tooth cleaning is carried out. After 1 h, the pH was measured using the special indicator MCloropHast pH 5.2–7.2 (Merck), and the ΔpH was determined. Table 1 shows the acid-formation potentials and the corresponding risk of caries based on the ΔpH values measured in this new saliva test.

**Table 1:** Categorisation of acid-formation potential and corresponding caries risk as a function of pH difference (ΔpH) in a novel saliva test (patent pending)

ΔpH	Risk class	Acid-formation potential	Risk of caries
≤ 0.5	1	low	In combination with low bacterial count of MS and/or LB, no caries risk
0.5–1	2	medium	In combination with medium to high bacterial counts of MS and/or LB, increased caries risk
≥ 1	3	high	In combination with high bacterial counts of MS and/or LB, high caries risk
≥ 1.2	4	very high	Very high caries risk

MS: *Streptococcus mutans*; LB: lactobacilli.

### Bacterial Tests

**Fig 1:**
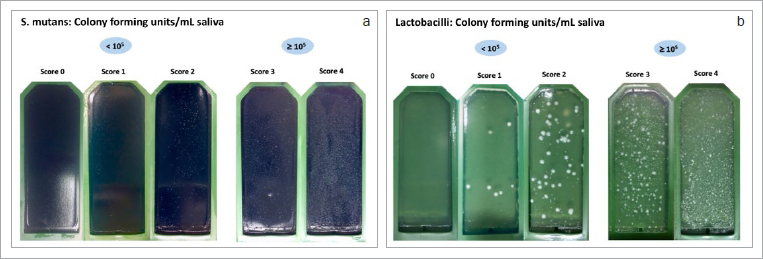
a. Classification of mutans streptococci CFU on a scale of 0–5 based on sample images from the present study. b. Classification of lactobacilli CFU on a scale of 0–5 based on sample images from the present study.

The bacterial count was determined using the caries risk test (CRT, Ivoclar Vivadent; Schaan, Liechtenstein), a double-sided dip-slide agar carrier with selective culture media for mutans streptococci (Mitis salivarius bacitracin agar) and lactobacilli (rogosa agar). After 48 h of incubation, colony-forming units (CFU) for mutans streptococci or lactobacilli were calculated per milliliter of saliva using a template supplied by the manufacturer. For a more precise evaluation, photographs were taken and sample images were assigned a score from 0 to 5 (Fig 1).

### Buffering Capacity

The buffering capacity was measured using the CRT (Ivoclar Vivadent) and the Saliva-Check BUFFER test (GC Germany; Bad Homburg, Germany). Results were divided into low, medium, and high buffering capacity.

### Saliva Microbiome

Before the dental examination, 2 ml of unstimulated saliva was taken from all participants and frozen at -80°C until microbiological analysis. The spitting method was used to collect unstimulated saliva that accumulated on the floor of the mouth and was spit into a pre-weighed test tube.^25^ Bacterial DNA was extracted using the QIAamp DNA Mini Kit (Qiagen; Hilden, Germany) as specified by the manufacturer’s instructions with partial modifications to the section on ‘Isolation of genomic DNA from Gram-positive bacteria’, ‘Appendix D’ (page 55), as previously described.^5^ Modifications consisted of using 180 µl lysozyme (20 mg/ml) during the first lysis at 37°C for 30 min, then adding 20 µl of proteinase K and 200 µl of buffer AL and lysing only for 10 min without the step of 95°C for 15 min, to avoid DNA degradation. DNA quantity and quality were analysed using a NanoDrop 1000 spectrophotometer (Thermo Fisher Scientific Germany; Braunschweig, Germany). The saliva microbiome was analysed using 16S rDNA next-generation sequencing. DNA was amplified using universal bacterial primers targeting the V4 region of the 16S rRNA gene (515F and 806R from Casporo et al^6^). Further descriptions of the microbiome sequencing technique are provided in Schoilew et al.^35^

### Statistical Analysis

Data were analysed using descriptive and microbiological statistics. Descriptive analyses were performed using Software R, version 3.6.3 ^30^ (R Foundation for Statistical Computing; Vienna, Austria) and included calculation of mean ± SD, range, medians and interquartile ranges. The Mann-Whitney U-test was carried out to determine statistically significant differences in ΔpH values, mutans streptococci and lactobacilli scores, GBI and PCR indices, and the buffering capacity between the groups. All p-values are purely descriptive. To evaluate the diagnostic quality of the test, receiver operating characteristic (ROC) curves were created and area under the curve (AUC) values were calculated.

Biostatistical analysis of the microbiological data and descriptive indices, such as alpha diversity (Shannon index), richness (number of ribosomal sequence variants [RSVs]), evenness (Pielou index), and dominance (Berger-Parker index), were carried out using the microbiome package. Permutational multivariate analysis of variance (PERMANOVA) was performed to determine statistically significant differences between the two groups. A DESeq2 analysis was also carried out to detect differences in RSV frequencies between the groups. Correlations between the microbiome indices, relative frequencies of RSVs, and quantitative clinical parameters were calculated using the Spearman correlation test, and p-values were adjusted using the Benjamini-Hochberg method. All microbiological analyses were carried out with R 3.1.4.^29^

## Results

### General Data

Table 2 shows the age, sex, and DMFT values of the two groups. Sex was fairly balanced (48% female, 52% male) and the mean age was 28.8 ± 10.9 years. Participants in the ‘caries’ group were statistically significantly older than those in the ‘healthy’ group (18–65 years vs 19–31 years; p = 0.01). In addition, there were statistically significant differences in GBI (p = 0.006) and PCR indices (p = 0.001) between the two groups, with higher values in the caries group. The mean GBI (%) value was 1.5 ± 2.9 in the ‘healthy’ group and 5.7 ± 6.8 in the ‘caries’ group. The mean PCR (%) was 28.4 ± 18.0 in the ‘healthy’ group and 46.4 ± 17.3 in the ‘caries’ group.

**Table 2:** Epidemiological and clinical data of study participants

		No. of samples	Sex		Age (years)	DMF(T)	D(T)	M(T)	F(T)
			female	male					
Group	Total	n = 50	48%	52%	28.8 ± 10.9 (18–65)	6.8 ± 8.1 (0–26)	2.0 ± 2.9 (0–11)	0.9 ± 2.2 (0–10)	3.9 ± 4.8 (0–15)
	Healthy	n = 25	44%	56%	24.5 ± 3.2 (19–31)	0.0 ± 0.0 (0–0)	0.0 ± 0.0 (0–0)	0.0 ± 0.0 (0–0)	0.0 ± 0.0 (0–0)
	Caries	n = 25	52%	48%	33.0 ± 13.9 (18–65)	13.6 ± 6.1 (1–26)	4.0 ± 3.0 (1–11)	1.8 ± 2.8 (0–10)	7.7 ± 4.1 (0–15)
Mann–Whitney U–Test (p)			0.575		0.01*				

DMF(T) values are presented as mean ± standard deviation (range).

### Acid-formation Potential

**Fig 2:**
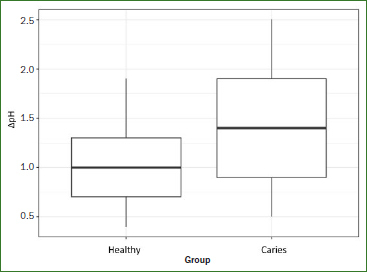
Comparison of ΔpH values (initial pH value minus pH value after 60 min of incubation in the nutrient medium of the new saliva test) between the ‘healthy’ (n = 25) and ‘caries’ (n = 25) groups. The ‘caries’ group had a statistically significantly higher ΔpH (p = 0.035).

**Fig 3:**
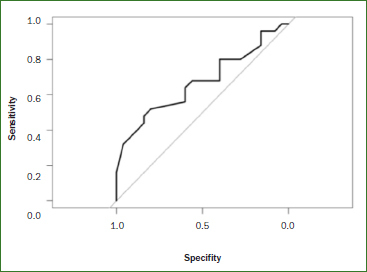
ROC curve of the measured ΔpH values of the ‘healthy’ (n = 25) and ‘caries’ (n = 25) groups. The AUC is the area under the ROC curve. The AUC value for ΔpH was 0.7 (1 = ideal).

The saliva pH at the beginning of the test was not statistically significantly different between the two groups (‘healthy’ group, 7.6; caries group, 7.5). After 1 h, the ‘caries’ group had a statistically significantly higher mean ΔpH value than did the ‘healthy’ group (1.4 vs 1.1; p = 0.035) (Table 3, Fig 2). The ROC curve was only marginally better than a diagonal curve. The AUC (which indicates how well the acid-formation potential of saliva predicts the occurrence of caries) was 0.67 (Fig 3). Based on the ΔpH, acid-formation potential was rated as low, medium, high or very high (Table 1). Differences in acid-formation potential between groups are shown as percentages. These differences were greatest among participants with very high acid-formation potential, whereas 64% of the participants of the ‘caries’ group were attributed to that group (Table 4).

**Table 3:** Descriptive data of ΔpH values in the ‘healthy’ and ‘caries’ groups

		Participants	ΔpH M	ΔpH SD	ΔpH med	ΔpH Q1–Q3	ΔpH range
Group	Healthy	n = 25	1.1	0.4	1.0	0.7–1.3	0.4–1.9
	Caries	n = 25	1.4	0.6	1.4	0.9–1.9	0.5–2.5
Mann-Whitney U-Test (p)		0.03536*					

M: mean; SD: standard deviation; med: median; Q1–Q3: interquartile range.

**Table 4:** Descriptive data of the acid-formation potential (ΔpH) of saliva in the ‘healthy’ group and the ‘caries’ group

		Participants	Acid-formation potential			
			Low	medium	high	very high
ΔpH			≤ 0.5	0.5–1	≥ 1	≥ 1.2
Group	Healthy	n = 25	8%	32%	20%	40%
	Caries	n = 25	4%	28%	4%	64%

### Bacterial Tests

Table 5 shows the results of the bacterial tests, scored from 0 to 4. No participants in the ‘caries’ group had a score of 0 or 1 for the distribution of mutans streptococcus, which indicates < 10^5^ CFU. In contrast, 17 participants (68%) of the ‘caries’ group scored 4, while only 4 participants (16%) of the ‘healthy’ group scored 4. This difference was statistically significant (p <0.001). In the ‘healthy’ group, 16 participants (64%) had a lactobacilli score of 0 and only 0–1 participants (0–4%) had a score of 3 or 4 (Table 5).

**Table 5:** Descriptive bacteria test scores for mutans streptococci and lactobacilli counts in the ‘healthy’ group and the ‘caries’ group

Mutans streptococci counts		Participants	Score				
			0	1	2	3	4
Group	Healthy	n = 25	12%	20%	32%	20%	16%
	Caries	n = 25	0%	0%	12%	20%	68%
Mann-Whitney U-Test (p)		2.71e-05*					
Lactobacilli counts		Participants	Score				
			0	1	2	3	4
Group	Healthy	n = 25	64%	28%	4%	4%	0%
	Caries	n = 25	16%	20%	12%	28%	24%
Mann-Whitney U-Test (p)		2.374e-05*					

### Buffering Capacity

The buffering capacity results are presented in Table 6. There were no statistically significant differences in buffering capacity between the two groups (p = 0.905).

**Table 6:** Descriptive data for buffering capacity in the ‘healthy’ and ‘caries’ groups

		Participants	Low	Medium	High
Group	Healthy	n = 25	4%	44%	52%
	Caries	n = 25	16%	28%	56%
Mann-Whitney U-Test (p)		0.9047			

### Saliva Microbiome

**Fig 4:**
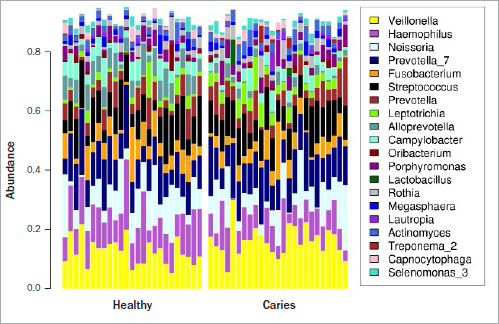
Bar chart of the 25 most common ribosomal sequence variants (genera) detected in saliva samples from the ‘healthy’ group (n = 25) and the ‘caries’ group (n = 25). The other genera were grouped together in the ‘other’ category.

A total of 954,209 ‘clean reads’ (cleaned measured values) were obtained from the 50 samples and a mock community with an average of 19106 (8120-33353) sequences per sample. A total of 855 RSVs were found. There were an average 115.32 (74-195) RSVs per saliva sample. In the ‘healthy’ group, the abundance of the genus *Alloprevotella* as well as the RSVs belonging to the genera *Alloprevotella* (rsv104), *Prevotella* (rsv242), *Campylobacter* (rsv341), and *Veillonella* (rsv658) was higher. In the ‘caries’ group, there was a significantly higher abundance of the genera *Fretibacterium* and *Lactobacillus* and the phyla Spirochaetes and Synergistetes, as well as the rsv816 belonging to the genus *Leptotrichia*. Figure 4 shows the most common RSVs of the saliva samples.

**Fig 5:**
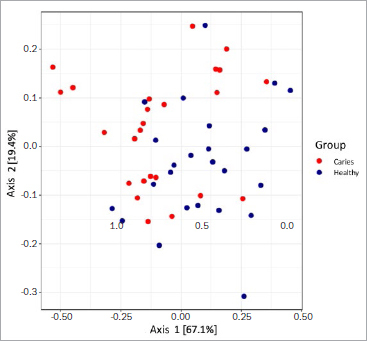
Comparison of the microbiome structure of the ‘healthy’ group (n = 25) and the ‘caries’ group (n = 25) using a principal component analysis based on Morisita-Horn distances. A heterogeneous distribution can be seen. 86.5% of the total variance is shown.

Figure 5 shows the structure of the saliva microbiome in the two groups according to principal coordinate analysis (PCoA) based on Morisita-Horn distances. Here, principal coordinate 1 represents 67.1% of the information and principal coordinate 2 represents 19.4%. Thus, the two principal coordinates together covered 86.5% of the total variance. Principal coordinate analysis revealed heterogeneity in both cohorts. Nevertheless, the samples of the ‘healthy’ group are separated from those of the ‘caries’ group, which is statistically validated by the PermaNova analysis (R2 value = 0.131, p = 0.003).

**Fig 6:**
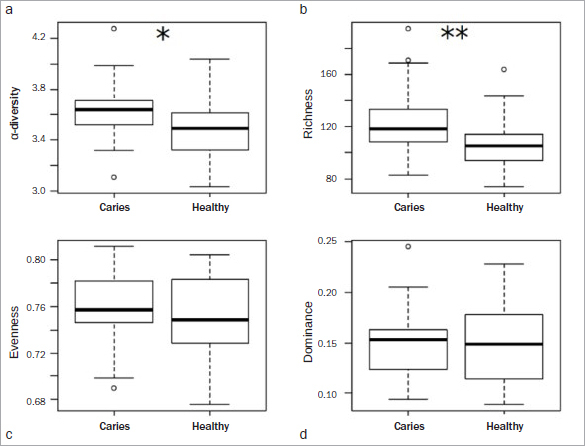
Box-and-whisker plots with medians and quartiles comparing the microbiome structure of the ‘healthy’ group (n = 25) and the ‘caries’ group (n = 25). a. Microbial α-diversity based on the Shannon index. b. Microbial richness, calculated as the number of observed RSVs. c. Microbial uniformity, based on the Pielou index. d. Microbial dominance, based on the Berger-Parker index. In (a) and (b), higher values were seen in the caries group. (c) and (d) are more balanced in the area of the median. However, the ‘healthy’ group shows a larger range of quartiles.

Furthermore, the saliva microbiome was more diverse (Fig 6a) and richer (Fig 6b) in the ‘caries’ group than in the ‘healthy’ group.

## Discussion

In the present study, we showed that the acid-formation potential (ΔpH) of saliva was significantly higher in the ‘caries’ group than in the ‘healthy’ group. The AUC values, which indicate how suitable the acid-formation potential of saliva is for predicting a caries-related change in the oral microbiome, was moderate (Fig 3), and bacterial tests discriminated well.

These results are underlined by our finding that numbers of mutans streptococci and lactobacilli were statistically significantly higher in the ‘caries’ group (Table 5). The use of saliva to determine or screen individuals’ caries risk remains controversial, and one-time saliva tests are known to be highly inaccurate, especially since other bacteria can be cultivated on the nutrient media. Because the results of one-time tests are often left to chance, it is advisable to carry out repetitions to ensure the diagnostic value.^10,12,32,36^ However, we detected a positive correlation between the number of microorganisms in plaque and saliva, which agrees with published findings.^23,37^ This indicates that the microbiological composition of saliva may reflect the microbial composition of plaque.

This pilot study explored a new approach to assessing or screening risk of caries, starting with assessing intraoral acid-formation potential. Caries development is widely thought to be caused by a shift in the homeostasis of host bacteria and intraoral bacteria due to changes in local environmental conditions.^21^ These environmental changes may include acid production by any number of microorganisms.^1^ The ΔpH values were statistically significantly higher in the ‘caries’ group after 1 h (p = 0.035, Table 3). In the ‘caries’ group, 64% of participants exhibited a very high acid-formation potential, supporting our earlier findings (Table 4). No differences in buffering capacity were detected between the two groups (Table 6), which corroborates published findings.^3^

Evaluation of the salivary microbiome revealed statistically significant differences in various genera and RSVs between the groups (Fig 4). Although ΔpH itself did not statistically significantly correlate to the microbiome, we were still able to detect a change in the microbiome between the two groups. Caries can be seen as a loss of protective function within the oral ecosystem, while the composition might be quite similar on the genus level. However, a few RSV and genera were statistically significantly different and the distance between the microbiomes moderate (R^2^ value = 0.131, p = 0.003), and an impact on the overall diversity and keystone genera was observed. In this context, the divergent acid-formation potential between the two groups might be potentially influence this change in the microbiome and therefore might contribute to the loss of function. Statistically significantly higher occurrences of *Lactobacillus, Fretibacterium,* Spirochaetes, Synergistetes and the RSV 816 (*Leptotrichia*) were detected in the ‘caries’ group. The higher incidence of lactobacilli is consistent with the CRT bacteria saliva test results described above (Table 5). Lactobacilli are not only marker bacteria,^4,40^ but are also predominantly associated with caries progression, as shown in previous studies.^1,7,18,24^ Thus, the results of the present study are consistent with previous reports. Our finding that *Leptotrichia* are more prevalent in the saliva microbiome of individuals with caries is consistent with the results of Ling et al^20^ and Qudeimat et al.^28^ In contrast, other studies have reported a higher occurrence of *Leptotrichia* in the saliva microbiome of individuals with no caries experience.^1,35^

Two genera of the Prevotellaceae family are enriched in the salivary microbiome of the ‘healthy’ group (genus *Alloprevotella*, RSV104, and RSV 242). Both rsv242 (*Prevotella*) and rsv104 (*Alloprevotella*) had a statistically significantly higher occurrence in the ‘healthy’ group. It was observed that different *Prevotella* species were present in individuals with and without caries and that the species were not evenly distributed between these two groups. In a study by Schoilew et al,^35^ *Prevotella* was one of 11 genera that occurred statistically significantly more frequently in individuals without caries experience compared to individuals with previous caries experience.

Another genus with a higher incidence in the ‘healthy’ group was *Veillonella* (RSV 658). According to Vesth et al,^41^ the importance of *Veillonella* in human infections is still uncertain, and this genus is generally considered to have low virulence. Nevertheless, *Veillonella* spp. have been associated with caries development.^28^ Aas et al^1^ showed that *Veillonella* spp. are present in intact enamel and carious lesions; however, *Veillonella* spp. are predominantly associated in stages within the carious process.

In this pilot study, α-diversity and richness were higher in the ‘caries’ group than in the ‘healthy’ group (Figs 5a and b), in agreement with previous findings by Yang et al.^42^ Earlier studies found a statistically significantly more variable microbiome in individuals with caries than in individuals without caries experience. In contrast, a recent study by Jiang et al^14^ showed no statistically significant differences in diversity and richness of the saliva microbiome in individuals with and without caries. It should also be noted that high diversity per se is not inevitably associated with a stable microbiome.^8,15^ Therefore, both diversity and stability of the microbiome are important for oral health.^8^

There are some limitations to the present study. The examinations were carried out by one dentist and the group sizes of 25 participants per group were quite small. Due to the preselection of the two groups, which was necessary to test the hypothesis, the assessment of the potential of ΔpH for risk prediction was not ideal. Therefore, the results should be interpreted with caution. Furthermore, saliva per se is subject to various intrinsic and extrinsic influences. For instance, the composition of saliva can be affected by biological fluctuations and circadian rhythms, particularly with regard to the microbiological composition. These influences may have affected our results. Besides, contradictory results from similar studies in that field may be related to variations in sampling, the degree of separation between and homogeneity within the cohorts being studied, as well as differences in DNA extraction and sequencing. Although current next-generation sequencing technology allows taxonomic profiling of microbiota at high resolution and depth, it must nevertheless be taken into account that the taxonomic assignment with 16S rDNA to genus and species level may be a challenge. To assign species the amplicon sequence variant is currently the most precise method. However, the species annotation is depending on the 16S region sequence and its variability.

Finally, the present investigation is a cross-sectional study in which an association between salivary parameters and pre-existing caries could be shown. If the test is to be used for preventive purposes, a subsequent study must be carried out to determine the extent to which it is suitable for predicting future caries development. This can be done in a prospective longitudinal study by calculating the validity (sensitivity and specificity) of the test.

## Conclusion

We showed that according to the differences found in the saliva microbiome, the acid-formation potential (ΔpH) of saliva was significantly higher in the ‘caries’ group than in the ‘healthy’ group. In terms of detecting a caries-related shift in the oral microbiome, overall, the acid-formation potential of saliva (ΔpH) discriminated to a moderate extent and bacterial tests discriminated well. Caries can be seen as a loss of protective function within the oral ecosystem, while the composition of the two groups might be quite similar on the genus level. We saw an impact on the overall diversity and a keystone genus. In this context, the different acid-formation potentials between the two groups might be a factor potentially influencing this change in the microbiome and therefore might contribute to the loss of function. Further studies must be carried out to determine the extent to which the two tests are suitable for predicting future caries development.
